# Association between levels of exposure to heavy metals and renal function indicators of residents in environmentally vulnerable areas

**DOI:** 10.1038/s41598-022-27292-7

**Published:** 2023-02-17

**Authors:** Jung-Yeon Kwon, Seungho Lee, Ulziikhishig Surenbaatar, Hyoun-Ju Lim, Byoung-Gwon Kim, Sang-Yong Eom, Yong Min Cho, Woo Jin Kim, Byeng-Chul Yu, Kwan Lee, Young-Seoub Hong

**Affiliations:** 1 Department of Preventive Medicine, College of Medicine, Dong-A University, 32, Daesin Gongwon-ro, Seo-gu, Busan, 49201 Korea; 2 Busan Environmental Health Center, Dong-A University, Busan, 49201 Korea; 3 Department of Preventive Medicine, College of Medicine, Chungbuk National University, Cheongju, 28644 Korea; 4 Department of Nano, Chemical and Biological Engineering, SeoKyeong University, Seoul, 02713 Korea; 5 Department of Internal Medicine and Environmental Health Center, Kang-Won National University, Chuncheon, 24341 Korea; 6 Department of Preventive Medicine, College of Medicine, Kosin University, Busan, 49267 Korea; 7 Department of Preventive Medicine, College of Medicine, Dongguk University, Gyeongju, 38066 Korea

**Keywords:** Environmental sciences, Risk factors

## Abstract

Abandoned metal mines and refineries are considered environmentally vulnerable areas owing to high levels of exposure to heavy metals. This study examined the association between heavy metal exposure and renal function indicators. We studied a total of 298 participants, of which 74 and 68 resided in low- and high-exposure abandoned metal mine areas, respectively, with 121 in the refinery area and 35 in the control area. Blood and urine samples were collected from the participants to analyze the levels of blood lead, cadmium, and creatinine and urinary cadmium, NAG, and β2-MG. The estimated glomerular filtration rate, which is calculated using the Chronic Kidney Disease Epidemiology Collaboration equation, was used for assessments. The study participants comprised more females than males, and their mean age was 70.3 years. The blood lead and cadmium as well as urinary cadmium levels were 2.12 μg/dL, 1.89 μg/L, and 2.11 μg/L, respectively, in the heavy metal-exposure areas, and 1.18 μg/dL, 0.89 μg/L, and 1.11 μg/L, respectively, in the control area. The odds ratio (OR) for exceeding the reference value showed that blood cadmium in the refinery area was 38 times higher than that in the control area. Urinary cadmium was seven times higher in the low-exposure abandoned metal mine area than in the control area. NAG showed a positive correlation with urinary cadmium in all areas. In the refinery area, correlations were observed between β2-MG and urinary cadmium levels and the eGFR and blood cadmium level; in the high-exposure abandoned metal mine area, correlations were observed between NAG, β2-MG, and the eGFR and blood cadmium. In this study, the association between Cd exposure and some renal function indicators was observed. This study’s findings and the obtained biological samples can serve as a basis for future molecular biological research.

## Introduction

In South Korea, a suspected incident of itai-itai disease occurred in an abandoned metal mine area in 2004, and the impact of abandoned metal mine contamination on local residents health has since become a social issue^1^. Since then, continuous research has been conducted to identify the health damages caused by environmental hazards and the correlation between environmental hazards and diseases^2–5^.

The mining and refining processes discharge large quantities of heavy metals into the environment. Specifically, mine drainage from abandoned metal mines contains large quantities of highly toxic heavy metals such as Lead (Pb), Cadmium (Cd), and Arsenic (As), which can contaminate the surrounding soil and lead to high heavy metal contents in farmland crops^6, 7^. Consuming contaminated crops can cause chronic disorders in humans and animals owing to the accumulation of heavy metals in the body^7, 8^. In South Korea, metal mining and refining processes have been actively conducted for decades, and the discharge of mining wastes has severely contaminated the surrounding soil and farmland^9, 10^. Consequently, areas around abandoned metal mines are especially vulnerable to contamination by heavy metals such as Cd, Pb, and As.

Refinery-caused soil contamination occurs owing to heavy metal dust being discharged through chimneys in the refining process and dust generated during the transportation of the ores^11^. According to a detailed soil survey conducted in 2007, Zn and Cd concentrations exceeded the contamination threshold within a radius of 500 m from a refinery, and those of Pb, As, Cu, and Ni exceeded the threshold within a radius of 800 m^3, 11^. The contamination level generally decreases as the distance from the refinery increases; thus, researchers have mostly investigated soil contamination within a radius of 2 km from a refinery. However, a few studies have also reported heavy metal contamination in soil up to 4 km from refinery chimneys, leading to a 2008 study analyzing soil contamination further than a radius of 3 km and a survey on the health effects of local residents^3, 11, 12^. A study that identified heavy metal concentrations in local residents and soil contamination levels according to the distance from the refinery reported that the heavy metal contamination level in soil increases as the distance from the refinery decreases. Researchers also reported that residents who lived close to the refinery had high heavy metal concentrations in the body^11, 13^.

Several studies have assessed exposure levels in vulnerable areas with identical exposure characteristics^7, 14–16^. However, few studies have comparatively analyzed the effects of each exposure levels heavy metals at the same time within two or more vulnerable areas.

This study analyzes and compares the heavy metal concentrations in residents of abandoned mine areas, a refinery area, a control area, and a general population group and evaluates its associated impacts on the functions of the kidney, which is a target organ of heavy metals in the human body.

## Materials and methods

### Study participants

Human participants were recruited from three environmentally vulnerable areas: a low-exposure abandoned metal mine area (LEAMM), high-exposure abandoned metal mine area (HEAMM), refinery area, and control area unaffected by contamination. The LEAMM and HEAMM areas were located in Goseong, South Gyeongsang province, with the refinery area in Seocheon, South Chungcheong province, and the control area in Gimhae, South Gyeongsang province. The 2 mine areas were classified into high and low exposure areas based on the results, especially soil contamination levels and environmental exposure status of residents from the “Planning and Pilot Study for 2nd Phase Health Effects Survey of Abandoned Metal Mines” conducted in 2012^17^. Residents of the HEAMM and the LEAMM lived within a radius of 2 km and 7 km from the mine pit, respectively. Residents of the refinery area lived within a radius of 4 km from the refinery. The control area was not affected by environmental pollution facilities. We explained the purpose and process of the study to the participants and then conducted a survey with residents who volunteered to participate. A total of 298 participants were recruited, including 74 from the LEAMM, 68 from the HEAMM, 121 from the refinery, and 35 from the control areas. From June to October 2021, blood and urine samples were collected from the participants. In addition, demographic characteristics, smoking, alcohol consumption, and disease history were obtained using questionnaire by trained investigators. We informed visitors at local district office of our study and the volunteers gave informed consent for use of personal information and permission of blood, urine sampling. Dong-A Institutional Review Board (IRB) is a bioethics review institution approved by the Ministry of Health and Welfare of Korea. This study was reviewed and approved by the Institutional Review Board of Dong-A University (IRB: 2-1040709-AB-N-01-202105-BR-002-08) and was conducted in accordance with the relevant guidelines and regulations.

### Blood and urine sampling

We collected blood samples from the participants’ veins using ethylenediaminetetraacetic acid (EDTA) vacutainers and serum separator tube (SST) vacutainers. After collection, we gently mixed the contents of the EDTA tube 8 to 10 times to prevent coagulation and roll mixed for 30 min in an agitator. The SST tube was left at room temperature for 30 min and centrifuged at 3,000 rpm for 10 min to separate the supernatant. The urine samples were collected and dispensed into 15 mL conical tubes. All samples were frozen until the analyses.

### Heavy metal analysis

Heavy metals were measured using an inductively coupled plasma mass spectrometer (ICP-MS). Blood Cd and Pb were analyzed using ICP-MS (NexION 200B, PerkinElmer, Waltham, MA, USA). Blood and urinary Cd was analyzed using ICP-MS (Agilent 7700x, Agilent Technologies, Santa Clara, CA, USA). Blood mercury (Hg) was analyzed using an automatic mercury analyzer (MA-3000, NIC, Tokyo, Japan).

Blood and urine samples were used for analysis after taking them out at room temperature and sufficiently stirring with a roll mixer for at least 30 min. The standard solutions of Pb, Cd, and Hg were serially diluted before use. In order to confirm the reliability of the analysis and verify the calibration curve, one point concentration of the calibration curve was checked for every 20 samples. Certified reference materials (CRM) were used for heavy metal analysis, and the test method was verified and reliability was secured. Blood ClinChek level 1, 2 (RECIPE Chemicals, Germany) was used for blood heavy metal analysis, and Urine ClinChek level 1, 2 (RECIPE Chemicals, Germany) was used as CRM for heavy metal analysis in urine. The blood sample (0.1 mL) was diluted with 4.8 mL of a mixed solution (ammonium pyrrolidinedithiocarbamate, ethanol, 25% tetramethylammonium hydroxide, Triton X-100, and nitric acid) and added to 0.1 mL of 1% nitric acid to measure blood Cd and blood Pb. For blood Cd and urinary Cd, we used 0.3 mL of the sample diluted with 2.7 mL of diluent (1-butanol, EDTA, Triton X-100, NH_4_OH). Blood Hg was measured by directly injecting 0.1 mL of the sample into the automatic mercury analyzer. Intra-day precision of all metals showed a RSD < 10%, and inter-day precision had a RSD between 0.3 and 15%.

### Analysis of renal function indicators of study participants

Urinary N-acetyl-β-glucosaminidase (NAG) was measured by a colorimetric assay using a NAG test kit (Nittobo Medical, Tokyo, Japan). Urinary Beta-2 microglobulin (β2-MG) was analyzed by an immunoturbidimetric assay using a β2-MG test kit (Roche, Basel, Switzerland). Blood creatinine was measured using the modified Jaffe method using a creatinine test kit (Roche). The estimated glomerular filtration rate (eGFR) was calculated by the equation of the Chronic Kidney Disease Epidemiology Collaboration (CKD-EPI, 2021) as follows: eGFR (mL/min/1.73 m^2^) = 142 × min (Scr/k,1)^α^ × max (Scr/k,1)^− 1.200^ × 0.9938^Age^ (× 1.012 if the individual was female). (k was 0.7 for females and 0.9 for males, α was − 0.241 for females and − 0.302 for males.).

### Statistical analysis

STATA Version 17.0 (College Station, Texas, USA) was used. The general characteristics were presented according to the exposed and the control area. Because of a right-skewed distribution, the heavy metal concentrations were converted to the natural log scale and presented the geometric mean with a 95% confidence interval (CI). Differences of heavy metal concentrations among the regions were analyzed by post-hoc test with the Bonferroni method. In case of creatinine adjusted urinary Cd, the reference range for creatinine was set to 0.3–3.0 g/L adopted from WHO guideline. The value below the range were imputed as the minimum of the range (0.3 g/L), and the value over the range were imputed as the maximum of the range (3.0 g/L). The association between renal function indicators and blood/urinary Cadmium was performed using Pearson correlation.

To compare the metal concentrations, we used data from two nationwide surveys: the Korea National Health and Nutrition Examination Survey (KNHANES) and the Korean National Environmental Health Survey (KoNEHS).

We used the reference value (RV95) of blood Cd in the KNHANES and urinary Cd in the KoNEHS as reference values for the Cd concentration of the study participants and performed a logistic regression analysis for each exposure characteristic. Two models were used. Model 1 was adjusted for sex, age, and smoking history, and Model 2 was, in addition to the variables of Model 1, adjusted for period of residence, alcohol consumption history, and intake of locally grown rice. The association of the renal function indicators and blood/urinary Cd concentrations were investigated by each area. The reference value for NAG was set at 11.5 U/L based on a previous study^18^. The reference value of β2-MG was set at 300 μg/L, which is a normal criterion provided by Medscape (https://www.medscape.com). The reference value of eGFR was set at 60 mL/min/1.73 m^2^, which was within the normal range provided by the National Kidney Foundation. Logistic regression analysis was conducted for the renal function indicators. We adjusted for sex, age, period of residence, and body-mass index (BMI) and presented the OR and CI of blood Pb and Cd, as well as urinary Cd for the renal function indicators. All analyses were conducted at a significance level under 5%.

## Results

### General characteristics

Table 1 shows the general characteristics of the study participants. We studied a total of 298 study participants, consisting of 36.6% males and 63.4% females. The composition of males and females in each survey area are as follows: 28 males (37.8%) and 46 females (62.2%) in the LEAMM area, 27 males (39.7%) and 41 females (60.3%) in the HEAMM area, 43 males (35.5%) and 78 females (64.6%) in the refinery area, and 11 males (31.4%) and 24 females (68.6%) in the control area. Accordingly, there were more females than males in every area. The average age of the participants was 70.3 years, which did not differ between the exposure and control areas. The average period of residence of all participants was 33.0 years, and in the LEAMM and HEAMM areas were 42.0 years and 39.0 years, respectively, which were longer than those of residents in the refinery and control areas. According to the survey for smoking and alcohol consumption, non-smokers (72.48% overall) and non-drinkers (49.65% overall) accounted for the largest groups in all areas. The residents in the LEAMM and refinery areas used water purifiers and tap water as their main drinking water sources, whereas those in the HEAMM and control areas used ground water in addition to water purifiers and tap water. Overall, 31.54% of residents consumed at least half of their rice from locally grown sources, and this proportion was highest in the HEAMM area at 64.71%.

**Table 1:** General characteristics of the study subjects.

	Total	Low-exposure abandoned metal mine	High-exposure abandoned metal mine	Refinery	Control	*p* value
	n (%)	n (%)	n (%)	n (%)	n (%)	
	298 (100)	74 (100)	68 (100)	121 (100)	35 (100)	
**Gender**						
Male	109 (36.6)	28 (37.8)	27 (39.7)	43 (35.5)	11 (31.4)	0.851^†^
Female	189 (63.4)	46 (62.2)	41 (60.3)	78 (64.6)	24 (68.6)	
**Age (year)**						
Mean ± SD	70.3 ± 10.1	70.3 ± 11.9	68.2 ± 12.7	71.5 ± 7.4	70.3 ± 7.7	0.206*
**Period of residence (year)**						
Mean ± SD	33.0 ± 25.1	42.0 ± 26.4	39.0 ± 23.8	25.6 ± 23.0	28 ± 23.6	< 0.001*
**Smoke**						
Current	21 (7.0)	3 (4.1)	7 (10.3)	9 (7.5)	2 (5.7)	0.762^†^
Past	61 (20.5)	18 (24.3)	11 (16.2)	24 (19.8)	8 (22.9)	
Never	216 (72.5)	53 (71.6)	50 (73.5)	88 (72.7)	25 (71.4)	
**Drink alcohol**						
Current	75 (25.2)	26 (35.1)	18 (26.5)	22 (18.2)	9 (25.7)	0.272^†^
Past	75 (25.2)	15 (20.3)	16 (23.5)	34 (28.1)	10 (28.6)	
Never	148 (49.6)	33 (44.6)	34 (50.0)	65 (53.7)	16 (45.7)	
**Drinking water**						
Purified water	164 (55.0)	57 (77.0)	39 (57.3)	52 (43.0)	16 (45.7)	< 0.001^†^
Tap water	112 (37.6)	17 (23.0)	21 (30.9)	69 (57.0)	5 (14.3)	
Ground water	22 (7.4)	–	8 (11.8)	–	14 (40.0)	
**Intake of locally grown rice**						
More than 50%	94 (31.5)	7 (9.5)	44 (64.7)	43 (35.5)	32 (91.4)	< 0.001^†^
Less than 50%	204 (68.5)	67 (90.5)	24 (35.3)	78 (64.5)	3 (8.6)	
**Job history**						
Mine	3 (1.0)	–	3 (4.4)	–	–	< 0.001^†^
Refinery	18 (6.0)	–	-	17 (14.0)	1 (2.9)	
No experience	277 (93.0)	74 (100.0)	65 (95.6)	104 (86.0)	34 (97.1)	
**eGFR (ml/min/1.73 m**^**2**^**)**						
≥ 60	278 (93.3)	69 (93.2)	66 (97.1)	111 (91.7)	16 (45.7)	0.531^†^
< 60	20 (6.7)	5 (6.8)	2 (2.9)	10 (8.3)	19 (54.3)	
**BMI (kg/m**^**2**^**)**						
< 18.5	7 (2.4)	1 (1.3)	4 (5.9	2 (1.6)	–	0.097^†^
18.5–24.9	163 (54.7)	36 (48.7)	40 (58.8)	61 (50.4)	26 (74.2)	
25–29.9	99 (33.2)	30 (40.5)	17 (25.0)	44 (36.4)	8 (22.9)	
≥ 30	29(9.7)	7(9.5)	7(10.3)	14(11.6)	1 (2.9)	

eGFR, estimated glomerular filtration rate; BMI, body mass index; SD, standard deviation.

*The *p* value indicates significance between subgroups using 1-way analysis of variance (ANOVA).

^†^The *p* value calculated by χ^2^chi-square test.

### Comparison of heavy metal concentrations

The geometric mean concentrations (95% CI) of blood Pb, Cd, and urinary Cd in all exposure areas were 2.12 (1.99, 2.25) μg/dL, 1.89 (1.75, 2.04) μg/L, and 2.11 (1.90, 2.35) μg/L, respectively.

By area, the blood Pb concentrations (95% CI) in the LEAMM, HEAMM, and refinery areas were 2.66 (2.52, 2.81), 1.77 (1.55, 2.02), and 2.04 (1.85, 2.25) μg/dL, respectively, and that in the control area was 1.18 (1.00, 1.40) μg/dL. Hence, concentrations were higher in the exposure areas than in the control area. Concentration of blood Hg was similar in all areas except the LEAMM area; however, the difference was not statistically significant (Table 2).

**Table 2:** Heavy metal concentrations by region of the study subjects.

	N	AM ± SD	GM (95% CI)	p25	p50	p95
**Blood Pb**^**†**^ **(μg/dL)**						
Low-exposure abandoned metal mine area	74	2.74 ± 0.85*	2.66 (2.52–2.81)	2.31	2.59	4.09
High-exposure abandoned metal mine area	68	2.05 ± 1.20*	1.77 (1.55–2.02)	1.11	1.85	4.01
Refinery area	121	2.39 ± 1.56*	2.04 (1.85–2.25)	1.36	1.91	5.70
Control area	35	1.33 ± 0.67	1.18 (1.00–1.40)	0.91	1.09	2.95
**Blood cd**^**†**^ **(μg/L)**						
Low-exposure abandoned metal mine area	74	1.55 ± 0.88	1.37 (1.22–1.53)	0.96	1.32	2.98
High-exposure abandoned metal mine area	68	2.56 ± 2.02*	1.93 (1.61–2.33)	1.05	1.95	7.02
Refinery area	121	2.59 ± 1.37*	2.27 (2.07–2.50)	1.73	2.29	5.30
Control area	35	0.96 ± 0.37	0.89 (0.78–1.02)	0.67	0.90	1.97
**Urine Cd**^**†**^ **(μg/L)**						
Low-exposure abandoned metal mine area	74	3.87 ± 3.13*	2.87 (2.36–3.49)	1.73	3.34	10.69
High-exposure abandoned metal mine area	68	3.18 ± 2.52*	2.46 (2.06–2.93)	1.37	2.54	6.90
Refinery area	121	2.34 ± 2.18	1.61 (1.37–1.89)	0.84	1.69	6.52
Control area	35	1.49 ± 1.31	1.11 (0.85–1.44)	0.60	1.00	4.63
**Urine Cd** **(μg/g Cr)**						
Low-exposure abandoned metal mine area	74	3.59 ± 2.51*	2.84 (2.40–3.36)	1.72	3.47	7.50
High-exposure abandoned metal mine area	68	3.94 ± 2.66*	3.14 (2.65–3.71)	1.91	2.94	9.27
Refinery area	121	3.53 ± 2.50*	2.86 (2.54–3.22)	1.88	2.95	8.67
Control area	35	1.55 ± 0.79	1.36 (1.14–1.63)	0.91	1.44	3.16
**Blood Hg**^**†**^ **(μg/L)**						
Low-exposure abandoned metal mine area	74	6.59 ± 4.01*	5.63 (4.94–6.42)	3.80	5.67	13.46
High-exposure abandoned metal mine area	68	4.32 ± 2.34	3.71 (3.22–4.27)	2.66	3.91	8.27
Refinery area	121	2.87 ± 1.98	2.38 (2.14–2.65)	1.48	2.34	6.62
Control area	35	3.00 ± 1.60	2.67 (2.26–3.16)	1.97	2.60	7.31

AM, arithmetic mean; SD, standard deviation; GM (95% CI), geometric mean (95% confidence interval).

*Indicates a significant difference between the exposed area and the control area.

^†^Indicates the significant differences among the exposed areas.

The geometric mean concentrations (95% CI) of blood Cd in the LEAMM, HEAMM, and refinery areas were 1.37 (1.22, 1.53), 1.93 (1.61, 2.33), and 2.27 (2.07, 2.50) μg/L, respectively, and that in the control area was 0.89 (0.78, 1.02) μg/L. Thus, the exposure areas showed higher concentrations than the control area, and the refinery area showed the highest blood Cd concentration. The geometric mean concentrations of urinary Cd in the LEAMM, HEAMM, and refinery areas were 2.87 (2.36, 3.49), 2.46 (2.06, 2.93), and 1.61 (1.37, 1.89) μg/L, respectively, and that in the control area was 1.11 (0.85, 1.44) μg/L. Thus, the exposure areas showed higher concentrations than the control area, and the LEAMM showed the highest urinary Cd concentration. The geometric means of urinary Cd adjusted with urinary creatinine values were also significantly higher in the exposure areas than the in control area (Table 2).

**Figure 1:**
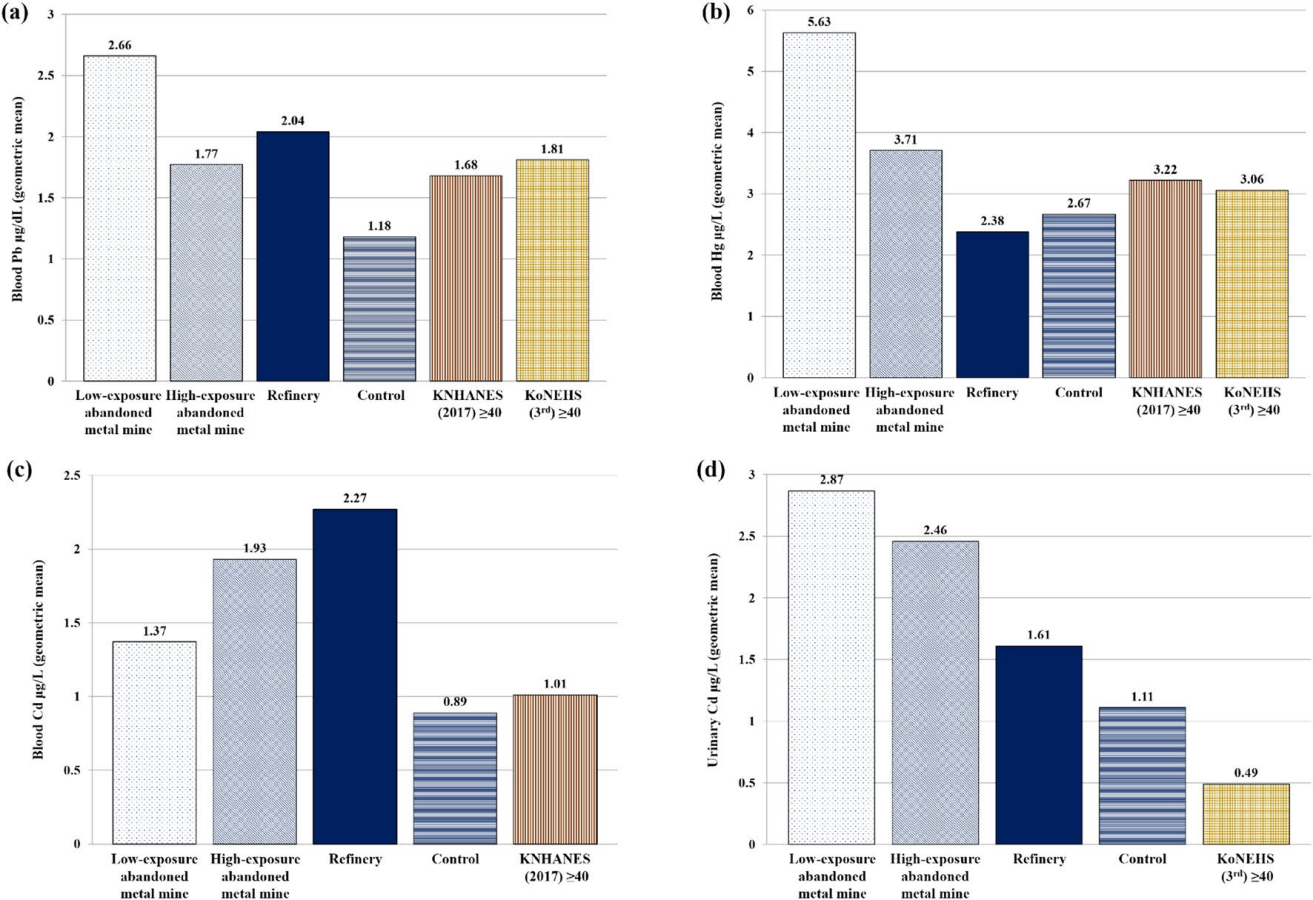
Comparisons of heavy metal concentrations between study subjects and the general population group. (**a**) Blood lead; (**b**) Blood mercury; (**c**) Blood cadmium; (**d**) Urinary cadmium. KNHANES: Korea National Health and Nutrition Examination Survey; KoNEHS: Korean National Environmental Health Survey.

Based on data of the 2017 KNHANES and the third KoNEHS, we calculated the geometric means of blood Pb, Hg, and blood and urinary Cd, and compared them with the concentrations of the study participants. The blood Pb concentrations in all exposure areas were higher than the KNHANES geometric mean (1.68 μg/dL). However, this concentration was higher than the KoNEHS geometric mean (1.81 μg/dL) in only the LEAMM and refinery areas, whereas that in the HEAMM area was lower (Fig. 1a). Blood Hg concentrations were higher in the LEAMM and HEAMM areas than the KNHANES and KoNEHS geometric means but lower in the refinery and control areas (Fig. 1b). All areas except the control area showed higher blood Cd concentrations than the KNHANES geometric mean (1.01 μg/L) (Fig. 1c). Urinary Cd concentrations were higher in all surveyed areas than the KoNEHS geometric mean (0.49 μg/L) (Fig. 1d).

### Correlation between renal function indicators and Cd

**Figure 2:**
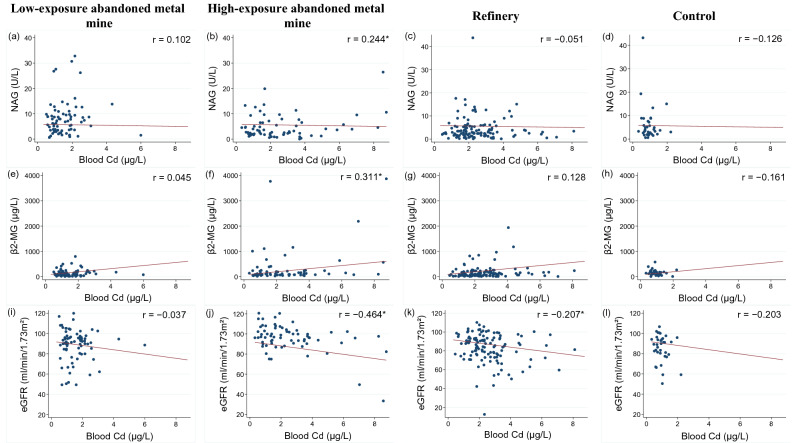
Correlation between blood Cd and renal function indicators. NAG: N-acetyl-glucosaminidase; β2-MG: Beta-2 microglobulin; eGFR: estimated glomerular filtration rate.

**Figure 3:**
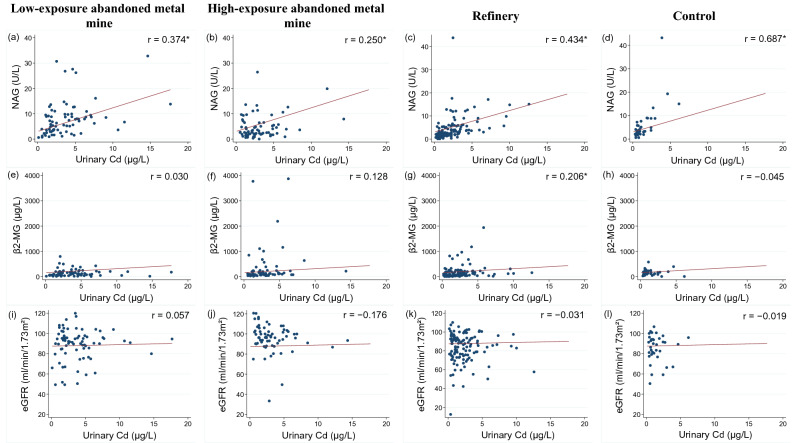
Correlation between urinary Cd and renal function indicators. NAG: N-acetyl-glucosaminidase; β2-MG: Beta-2 microglobulin; eGFR: estimated glomerular filtration rate.

Figure 2 and 3 are shown the correlation between the renal function indicators and blood/urinary Cd.

Blood Cd and NAG showed a statistically significant (p = 0.045) positive correlation (r = 0.244) in the HEAMM area, while no statistically significant correlation was observed for the other areas (Fig. 2a–d). Blood Cd and β2-MG levels showed a positive correlation (r = 0.311) in the HEAMM area, but the correlation was weak in the remaining areas (Fig. 2e–h). The HEAMM area showed the highest negative correlation between blood Cd level and eGFR (r = − 0.464) and the refinery area showed a lower negative correlation (r = − 0.207). However, the LEAMM and control areas showed either an extremely low correlation or no statistically significant correlation (Fig. 2i–l).

Urinary Cd and NAG levels showed a statistically significant positive correlation in all areas (Fig. 3a–d). The control area showed the highest correlation (r = 0.687, *p* < 0.001), and the HEAMM area showed the lowest correlation (r = 0.250, *p* = 0.040). The correlation between urinary Cd and NAG levels was higher in the refinery area than in the LEAMM and HEAMM areas. β2-MG and urinary Cd levels showed a statistically significant (*p* = 0.024) positive correlation (r = 0.206) in the refinery area, and a weak correlation in the remaining areas (Fig. 3e–h). The correlations between urinary Cd level and eGFR were weak or not statistically significant in all areas (Fig. 3i–l).

### Exceeding reference ratios of the values

Table 3 shows a comparison of the ORs of exceeding the reference value (RV95) for Cd with the general population by area.

**Table 3:** Odds ratios of exceeding the reference values for cadmium by regions.

	Model 1		Model 2	
	OR (95% CI)	*p *value	OR (95% CI)	*p *value
**Blood Cd** **(μg/L)**				
Low-exposure abandoned metal mine	5.89 (1.26, 27.63)	0.024	5.42 (1.14, 25.79)	0.034
High-exposure abandoned metal mine	23.11 (4.95, 107.81)	< 0.001	21.39 (4.33, 105.65)	< 0.001
Refinery	35.66 (7.94, 160.26)	< 0.001	38.29 (8.24, 177.90)	< 0.001
Control	1.00		1.00	
**Urinary Cd** **(μg/L)**				
Low-exposure abandoned metal mine	8.46 (3.33, 21.50)	< 0.001	7.08 (2.73, 18.39)	0.041
High-exposure abandoned metal mine	6.03 (2.40, 15.17)	< 0.001	2.87 (1.05, 7.89)	< 0.001
Refinery	2.48 (1.08, 5.71)	0.032	1.82 (0.75, 4.39)	0.184
Control	1.00		1.00	

OR (95% CI), odds ratio (95% confidence interval); Reference value of blood Cd, 1.94 μg/L (The Korean National Health and Nutrition Examination Survey (KNHANES) 2017, p95); Reference value of Urinary Cd, 1.75 μg/L (Korean National Environmental Health Survey (KoNEHS) 3rd survey, p95).

Model 1: Adjusted for gender, age, and smoking.

Model 2: Adjusted for gender, age, smoking, period of residence, alcohol drinking, intake of locally grown rice.

The ORs (95% CI) for blood Cd in the LEAMM, HEAMM, and refinery areas in Model 1 were higher than the control area by 5.89 times (95% CI 1.26, 27.63), 23.11 times (95% CI 4.95, 107.81), and 35.66 times (95% CI 7.94, 160.26), respectively, and those in Model 2 were higher by 5.42 times (95% CI 1.14, 25.79), 21.39 times (95% CI 4.33, 105.65), and 38.29 times (95% CI 8.24, 177.90), respectively. The OR (95% CI) for urinary Cd in the LEAMM, HEAMM, and refinery areas in Model 1 were higher than the control area by 8.46 times (95% CI 3.33, 121.50), 6.03 times (95% CI 2.40, 15.17), and 2.48 times (95% CI 1.08, 5.71), respectively, and those in Model 2 were higher by 7.08 times (95% CI 2.73, 18.39), 2.87 times (95% CI 1.05, 7.89), and 1.82 times (95% CI 0.75, 4.39), respectively. The ORs of exceeding the reference value for blood and urinary Cd were more significant in Model 1 than in Model 2.

### Associations between renal function indicators and heavy metals

To examine the relationships between renal function indicators and heavy metals, we performed a logistic regression analysis on the values greater or less than the reference values of NAG, β2-MG, and eGFR, which are renal function indicators (Table 4). We did not distinguish between the areas. and used sex, age, period of residence, and BMI as the adjustment variables. Then, we investigated the relationship with log-transformed heavy metal concentrations. As the urinary Cd concentration increased, the probability of exceeding the reference value of NAG became 5.27 times higher (OR 5.27, 95% CI 2.71, 10.27), and BMI (*p* < 0.05) showed a significant correlation with NAG level. In addition, as the blood Cd concentration increased, the probability of exceeding the reference value of β2-MG was 2.37 times higher (OR 2.37, 95% CI 1.15, 4.90), and age (*p* < 0.05) showed a significant correlation with β2-MG level. As the concentration of blood Cd increased, the probability of falling below the reference value of eGFR became 4.08 times higher (OR 4.08, 95% CI 1.54, 10.77).

**Table 4:** Odds ratios of heavy metal levels for renal function indicators.

	OR	95% CI	*p *value
**NAG**			
Blood Pb	1.366	(0.582, 3.203)	0.474
Blood Cd	0.678	(0.316, 1.454)	0.318
Urinary Cd	5.272	(2.707, 10.267)	< 0.001
**β2-MG**			
Blood Pb	0.400	(0.182, 0.879)	0.023
Blood Cd	2.375	(1.152, 4.896)	0.019
Urinary Cd	1.506	(0.948, 2.391)	0.083
**eGFR**			
Blood Pb	0.714	(0.252, 2.023)	0.527
Blood Cd	4.077	(1.543, 10.769)	0.005
Urinary Cd	0.572	(0.327, 1.002)	0.051

OR, odds ratio; 95% CI, 95% confidence interval; NAG, N-acetyl-glucosaminidase, β2-MG, Beta-2 microglobulin; eGFR, estimated glomerular filtration rate.

Criteria for NAG is above 11.5 U/L; for β2-MG is above 300 μg/L; for eGFR is below 60 ml/min/1.73 m^2^.

[Note] The variables in the first column are renal function indicators adjusted for gender, age, period of residence, body mass index; heavy metal concentrations were log transformed.

## Discussion

This study is an environmental epidemiologic study based on exposure areas, in which we evaluated heavy metal exposure levels and their impact on renal functions. This was performed by directly comparing and analyzing the heavy metal exposure level and renal function indicators for residents in the LEAMM, HEAMM, and refinery areas, which are typical environmentally vulnerable areas in terms of exposure to heavy metals.

The blood Pb concentrations were higher in all exposure areas than in the control area at a statistically significant level (*p* < 0.05). In a study on residents of 38 abandoned metal mine areas in South Korea between 2008 and 2011^19^, the blood Pb level was 2.87 μg/dL, which is higher than the concentrations of both the general population group as well as the LEAMM (2.66 μg/dL) and HEAMM (1.77 μg/dL) areas of this study. In a study on residents of abandoned metal mine areas from 2013 to 2017^20^, the blood Pb concentration was 2.27 μg/dL; although this is lower than the result of the survey conducted from 2008 to 2011, it is, nonetheless, comparatively high. The blood Pb in the LEAMM area was higher than that reported by Moon et al.^20^, but the concentration in the HEAMM area was lower. This suggests that Pb contamination is higher in the LEAMM area than in the HEAMM area. The blood Pb concentration of adults living in Pb–Zn mine areas in Nasawara, Nigeria was 3.1 μg/dL^21^, which is higher than that of the LEAMM and HEAMM areas of this study; this is likely because mining activities in Nigeria are ongoing. The average blood Pb concentration of the residents in the refinery area in this study was 2.04 μg/dL, which is lower than that reported in similar prior studies^3, 13, 22^ but higher than the KNHANES (1.68 μg/dL) and KoNEHS (1.81 μg/dL) concentrations. The blood Pb concentrations of residents in a refinery area in Legnica, Poland was high, at 0.766 ± 0.14 μg/ml; five years later it decreased to 0.44 ± 0.14 μg/ml, but remained at a high level^23^. This is because the village was located only 0.9 km away from the refinery and the contamination caused by the refinery was severe. Because the refinery in our study is currently not operational, the exposure levels of the area’s residents have decreased compared to the past, but they remain at a high level and require continuous management.

Blood Hg showed similar concentrations in all areas except for in the LEAMM area. Furthermore, the blood Hg concentrations were lower in the residents of the refinery and control areas compared to the KNHANES and KoNEHS results. In this study, blood Hg was not correlated with environmental exposure factors.

In this study, the blood Cd concentration was 1.5 to 2.5 times higher in the LEAMM (1.37 μg/L), HEAMM (1.93 μg/L), and refinery (2.27 μg/L) areas than in the control area (0.89 μg/L) (*p* < 0.001). The blood Cd concentration was also higher in the exposure areas than the KNHANES (1.01 μg/L). In a study by Gil et al.^24^ the blood Cd concentration of a group affected by a mine spill in the southwestern region of Spain was 0.19 μg/L, which was higher than that of the unaffected group (0.14 μg/L). Although the concentration was lower than that of this study, the mine-affected group having shown a high blood Cd concentration is similar to the trend of this study. In a study by Moon et al.^20^ the geometric mean concentration of blood Cd was 1.42 μg/L; in comparison, the LEAMM and HEAMM areas showed a lower and higher concentration, respectively. In a 2007 study on residents of abandoned metal mine areas with high soil contamination levels in Busan and South Gyeongsang province^8^ the blood Cd concentration of the residents was 1.93 μg/L, which is similar to the concentration in the HEAMM area. In a 2017 study on residents of a refinery area^3^ the blood Cd concentration was 2.75 μg/L; although the blood cd concentration of the refinery area residents in the present study decreased compared to 2017, it is nonetheless higher than that of the general population group.

The urinary Cd concentrations of residents were 2.8 and 2.2 times higher in the LEAMM (2.87 μg/L) and HEAMM (2.46 μg/L) areas, respectively, than those of residents in the control area (1.11 μg/L) (*p* < 0.001). The Residents Health Effect Survey was conducted in 2014 in the HEAMM area of this study; the geometric means of blood and urinary Cd in local residents were 5.33 and 5.31 μg/L, respectively, which were higher than those of this study^25^. In this survey, the geometric means of blood and urinary Cd in the residents of the HEAMM area were 1.93 μg/L and 2.46 μg/L, respectively, representing a decrease from 2014 but remaining at a high level. This indicates that residents of the HEAMM area are continuously exposed to Cd. The urinary Cd concentration was higher in the refinery area (1.61 μg/L) than the control area (*p* = 0.001) in this study. The mean urinary Cd concentration of the residents of a refinery area in 2021 was 1.32 µg/L^22^, which is lower than that of the refinery area residents in this study. Although the operation has stopped in the refinery area of this study, the Cd exposure level is high, and it cannot be ignored. Briki et al.^26^ measured the urinary Cd concentrations in residents of mine and refinery areas in Hezhang, China as 0.33 ± 0.43 and 0.16 ± 0.99 μg/L, respectively and another study in China measured the urinary Cd concentrations of mine and refinery area residents as 5.7 ± 3.1 and 5.5 ± 3.5 μg/L, respectively^27^. Both these studies showed higher urinary Cd concentrations near the mine areas than near the refinery areas, which is similar to the results in our study. The urinary Cd concentration adjusted by creatinine was higher at a statistically significant level in the LEAMM (2.84 μg/g creatinine), HEAMM (3.14 μg/g creatinine), and refinery (2.86 μg/g creatinine) areas than in the control area (1.36 μg/g creatinine) and the KNHANES (0.68 μg/g creatinine) (*p* < 0.001). The urinary Cd concentrations in the exposure areas of this study are also higher than those reported by Moon et al.^20^ (1.66 μg/g creatinine) and Kim et al.^28^ (1.53 μg/g creatinine). According to previous studies^8, 16^, blood or urinary Cd concentrations will increase as the period of residence in an abandoned metal mine area increases. Although these studies did not present their results, they indicated that the blood or urinary Cd concentrations increased with the period of residence near an abandoned metal mine area, and that this trend was higher for urinary Cd. The correlations between blood and urinary Cd concentrations and the period of residence were not statistically significant in the refinery area.

The ORs of the Cd concentration in the body exceeding RV 95 of the general population group were compared between the areas. In addition to age, sex, and smoking history, we adjusted for period of residence, alcohol consumption, and intake of locally grown rice, as these can affect heavy metal concentrations in the body^16^. The ORs of exceeding the reference values of blood and urinary Cd in the exposure areas were higher than those in the control area at a statistically significant level (*p* < 0.05). The OR for blood Cd was higher in the refinery area than the abandoned metal mine areas, but that of urinary Cd was the opposite. These results confirm that Cd exposure is considerably higher in the exposure areas than in the control area. For blood Pb concentrations, we could not calculate the ORs in the exposure areas because they did not exceed the reference value of the control area.

Cd measured in blood and urine reflects the long-term Cd exposure level. If the increased Cd level in the body accumulates in the kidney, thus damaging the renal tubules, it increases the activity of urinary NAG and the excretion of β2-MG. Therefore, NAG and β2-MG are useful renal function indicators^16, 29, 30^. Thus, because the activity of urinary NAG shows a strong correlation with urinary Cd, it is often used as an indicator of the impact of Cd on the kidneys. Additionally, an increase in the excretion of urinary β2-MG is considered as an important indicator of renal damage caused by Cd^29, 31–33^. Therefore, several epidemiologic studies use urinary Cd as an environmental exposure indicator and NAG and β2-MG as indicators of renal tubular function disorders to investigate the impacts of environmental Cd exposure^33–35^. Moreover, because tubule damage caused by chronic exposure to low Cd concentrations may lead to typical forms of kidney damage, such as a decreased glomerular filtration rate (GFR), GFR is also used as an indicator of kidney damage resulting from Cd exposure^5, 36^. Accordingly, this study analyzed renal function indicators and examined their relationships with Cd. Kim et al.^13^ compared NAG activity and found that they were higher in an exposure group residing near a refinery than the control group at a statistically significant level (*p* = 0.019). In this study, the NAG activity was higher in the LEAMM area than in the control area, but this difference was not statistically significant. The highest β2-MG concentration was found in the HEAMM area, but the difference was not statistically significant compared to that in the control area, and the other exposure areas did not show a significant difference compared to the control area. In previous studies that investigated renal function indicators in exposure and control areas^16, 28^, NAG and β2-MG were higher in the exposure area than the control area; however, the difference was not statistically significant. These findings are similar to the results of this study in the abandoned metal mine areas (results not presented).

NAG showed a high positive correlation with urinary Cd concentration in both the exposure areas and the control area of this study. Without distinguishing between the areas, NAG and urinary Cd levels showed a high positive correlation (r = 0.40, *p* < 0.001), which was similar to the results of Kawada et al.^37^. In previous studies^2, 33, 38^, urinary Cd and β2-MG levels showed a significant correlation. In this study, the correlation between urinary Cd and β2-MG was high in the refinery area and low in the other areas. Blood Cd level and eGFR showed a significant negative correlation in the refinery and HEAMM areas, where the blood Cd concentrations were high. This is similar to the results of a previous study results, in which the value of eGFR decreased as the Cd concentration increased^39^.

A 2010 study in Thailand on Cd exposure and renal dysfunctions^33^ reported that urinary Cd had notable correlations with NAG and β2-MG but no significant relationship with eGFR. These results are similar to those shown in the refinery area of this study. In a 2021 study on renal dysfunctions in Myanmar, urinary Cd showed a positive correlation with β2-MG and a negative correlation with eGFR in Cd-exposure areas. A 2017 study on Cd and renal function indicators in South Korean adults^2^ also reported that urinary Cd was positively correlated with NAG and β2-MG and negatively correlated with eGFR. In this study, urinary Cd concentration showed a positive correlation with NAG in all areas but showed a positive correlation with β2-MG in only the refinery area. The correlation between urinary Cd and eGFR was low or not statistically significant in all areas, whereas the correlation between blood Cd and eGFR was high in some exposure areas.

Urinary NAG activity is measured as an indicator of renal toxicity caused by Pb. According to a study on Pb-handling workers^40^, urinary NAG activity has a significant correlation with blood Pb level. However, in this study, the correlation was weak and statistically insignificant (results not presented).

According to a study evaluating the relationship between renal tubular damage and urinary Cd concentration^2^, the prevalence of renal tubular damage, which is defined as high NAG and β2-MG levels, increased with the urinary Cd concentration. According to another study evaluating the relationship between impaired kidney function and urinary Cd concentration^41^, urinary Cd is significantly related to impaired kidney function (*p* = 0.028) and increase of urinary Cd is increased prevalence of impaired kidney function. In this study, the OR of exceeding the NAG reference value (NAG > 11.5 U/L) were positively correlated with the urinary Cd (*p* < 0.001). As the blood Cd concentration increased, the OR of β2-MG exceeding the reference value (β2-MG > 300 μg/L) and eGFR being less than the reference value (eGFR < 60 mL/min/1.73 m^2^) increased at a statistically significant level (*p* < 0.05).

According to the Environmental Statistics Yearbook^42^, soil in mines and refinery areas are more contaminated than the general land. Since the refinery was completely shut down in 1989, smelting and production processes are not in operation. Besides, the central government and local governments conducted a detailed survey, established a comprehensive plan for purification, and has been conducting land purification since 2012. In the case of agricultural land, purification was completed using the soil improvement method, and non-farming land was cleaned using the soil washing method. Despite the implementation of the soil pollution prevention project, heavy metals accumulated in the soil seem to remain for a long time.

It was reported that cultivated soils around abandoned mines had higher content of heavy metals than in general field^7^. Likewise, the concentrations of arsenic, copper, and lead in the soil near the refinery exceeded the standard for soil contamination^11^. Soil contamination can lead to heavy metal accumulation in crops and consequently affect human exposure of nearby residents. The study subjects also lived near abandoned mines and refinery and would affected for a long time. Thus, the biomonitoring levels of the heavy metals among the subjects were high.

Seven percent of all subjects had the past occupational exposure (work experience in a mine or refinery), and the duration of the exposure was more than 30 years. But there was no difference in heavy metal levels according to the occupational history. Therefore, the heavy metal concentrations in subjects are not related to occupational exposure.

Based on the results of this study, we confirmed that heavy metal concentrations were high in residents of the environmentally vulnerable areas. We compared and evaluated the heavy metal exposure levels of residents in high-risk areas with different exposure characteristics. This study has the following limitations: measurements were not performed repeatedly because it was a cross-sectional study, and the age of the study participants was high owing to the local demographic characteristics. Nevertheless, as heavy metals accumulate in the body over a long period owing to their long half-life, they were reflected in the environmental exposure indicators of the study areas.

## Conclusion

In this study, we evaluated the heavy metal concentrations of residents living in vulnerable areas and observed the relationship between Cd exposure and some renal function indicators. We expect the results of this study and the obtained biological samples to serve as references for future molecular biological studies on metabolites, proteomes, and epigenomes.

Heavy metals accumulated in the body remain for a long time under the continuous exposure from the contaminated environment. Thus, continuous monitoring on the health effects in the contaminated area is required.

## Data Availability

All data generated or analyzed during the current study are available from the corresponding author on reasonable request.
